# From Gestational Age–Specific Serum Creatinine Trajectories to an Interactive Clinical Interpretation Tool in Level IV NICU Infants

**DOI:** 10.21203/rs.3.rs-10296400/v1

**Published:** 2026-07-16

**Authors:** Jennifer Rumpel, Sofia Perazzo, Andrew South, Elizabeth Bonachea, Mona Khattab, matthew Gillen, Joseph Asante, Semsa Gogcu, Daniel Liu, Michelle Starr, Thomas Nienaber, Cara Slagle, Clare Brown, Matthew Harer, Tasnim Najaf, Rachel Han, Patricio Ray, Jonathan Bona, Mario Schootman, Corey Nagel

**Affiliations:** Arkansas Children’s Hospital; Children’s National Hospital; Atrium Health Levine Children’s Brenner Children’s Hospital; Nationwide Children’s Hospital and The Ohio State University; Wake Forest Baptist Health; Indiana University School of Medicine; Cincinnati Children’s Hospital Medical Center; Riley Hospital for Children at Indiana University Health; University of Wisconsin School of Medicine and Public Health; Washington University School of Medicine; Indiana University School of Medicine; University of Arkansas for Medical Sciences

## Abstract

**Objective:**

To characterize gestational age (GA)-specific serum creatinine (SCr) trajectories among level IV neonatal intensive care unit (NICU) infants and develop an interactive interpretation tool.

**Study Design:**

We analyzed ADVANCE database records linking kidney-specific data with Children’s Hospitals Neonatal Consortium clinical data from ten level IV NICUs (2010–2022). Infants with ≥ 1 SCr measurement (DOL 1–365) were included. GA-specific trajectories were generated for DOL 1–28 and week of life 1–16; an interactive tool was developed for clinical use.

**Result:**

Among 34,306 infants contributing 390,612 SCr measurements, SCr trajectories differed by GA. Peak SCr ranged from 0.93 mg/dL (IQR 0.80–1.10) at 22–24 weeks’ GA (DOL 4) to 0.76 mg/dL (IQR 0.65–0.90) among term infants (DOL 2), declining to a similar nadir (~ 0.20–0.23 mg/dL) across GA groups.

**Conclusion:**

SCr trajectories vary by GA and postnatal age in this population. These trajectories and the accompanying tool provide context for interpreting SCr values.

## Introduction

Serum creatinine (SCr) is the most commonly used biomarker to assess kidney function in infants and is routinely used to identify acute kidney injury (AKI) and guide medication dosing and fluid management. However, interpretation of SCr levels in infants is particularly challenging. At birth, SCr reflects maternal creatinine levels. Neonatal kidney function is initially immature; glomerular filtration rate increases rapidly over the first weeks of life as nephrons mature.^1,2,3^ These physiologic factors result in dynamic postnatal changes in SCr concentrations, particularly among preterm infants.^4,5^ Additionally, nephrogenesis continues until approximately 34 to 36 weeks’ gestation,^6,7^ resulting in substantial developmental differences in nephron maturity and renal functional reserve across gestational age (GA) groups. Accordingly, interpretation of neonatal SCr requires consideration of both GA and postnatal age.^8,9^

Despite the importance of SCr in clinical decision-making, available data describing neonatal SCr patterns remain limited. Foundational studies among healthy term^10^ and preterm^11^ infants have provided important reference values for these populations; however, prior studies have included small, single-center cohorts or have focused on relatively healthy term infants,^15^ often excluding critically ill or medically complex populations,^12,13,14^ and most report cross-sectional reference values rather than longitudinal trajectories, which may inadequately capture the rapid postnatal changes in SCr that occur across the first weeks of life.^5^ In current practice, infants cared for in level IV NICUs frequently have congenital anomalies, genetic abnormalities, or significant medical comorbidities, or require surgical intervention, and SCr patterns in these high-acuity populations may differ from those observed in healthier cohorts.^17^ Data describing SCr values in very preterm infants (< 32 weeks’ gestation) are particularly limited — a critical gap given the importance of SCr-based clinical decisions in this vulnerable population. Infants in level IV NICUs often have highly complex medical needs and prolonged hospital stays; without gestational age (GA)-specific longitudinal context, clinicians may have difficulty distinguishing expected physiologic changes in SCr from potentially abnormal kidney function — a distinction that is critical for the identification of AKI, medication dosing, and broader clinical decision-making in the NICU. Prior GA-specific, longitudinal descriptions of SCr patterns in extremely preterm and high-risk NICU infants have typically been limited to small cohorts with restricted observation windows. Developing GA-specific longitudinal SCr trends in level IV NICU patients therefore remains an important unmet need.

The objective of this study was to characterize longitudinal SCr trajectories across GA and postnatal age using a large multicenter cohort of infants hospitalized in level IV NICUs. We generated GA-specific trajectories using both daily and weekly postnatal summaries to provide clinically relevant benchmarks for SCr interpretation in high-acuity neonatal populations. These trajectories should not be interpreted as normative values for healthy infants, but rather as trajectories derived from critically ill infants with diverse comorbidities cared for in contemporary level IV NICUs.

To facilitate clinical application, we also developed an interactive tool to support interpretation of individual SCr values relative to these trajectories. Providers can use the tool to compare a patient’s SCr value to these GA- and postnatal age-specific trajectories, which may help distinguish expected physiologic patterns from abnormal values.

## Methods

### Longitudinal Trajectory Analysis

We used the ADVANCE relational database, which links kidney-specific electronic health record data with detailed NICU clinical data from ten level IV NICUs located in 9 states (AR, DC, NC, OH, WI, TX, IN, GA, MO) from 2010 to 2022.^18^ Clinical data were collected across participating sites through the Children’s Hospitals Neonatal Database (CHND; Children’s Hospitals Neonatal Consortium, Dover, DE; www.thechnc.org) and collated in a standardized format for integration into ADVANCE.^19^ Data abstraction procedures within the CHND have been described previously and include prospective, ongoing training in clinical definitions alongside systematic auditing to ensure reliability and validity at each center. Participating CHNC sites share several common characteristics: greater than 50 NICU beds, more than 400 neonatal admissions annually, a predominantly outborn population (> 70%), and availability of in-house pediatric and surgical subspecialty services.

Infants were eligible if they had at least one SCr measurement obtained between day of life (DOL) 1 and 365 during their NICU hospitalization. Eligibility was intentionally broad — AKI, mortality, prematurity, and congenital anomalies were not exclusionary — to reflect the complex, high-acuity population typically admitted to level IV NICUs. Given the descriptive nature of the study, analysis centered on summary statistics (medians and IQRs) rather than inferential testing.

Because infants frequently undergo multiple laboratory assessments within a single day, an infant-day summary dataset was constructed to avoid within-day pseudoreplication, such that each infant contributed a single value per DOL, defined as the median SCr measured on that day. For weekly analyses, an infant-week dataset was derived independently from the raw SCr data, such that each infant contributed a single value per week of life (WOL), defined as the median of all raw SCr measurements obtained during that week. Median aggregation was selected to reduce the influence of transient outlier measurements and variability related to timing of laboratory acquisition during periods of acute clinical instability.

**Figure 1:**
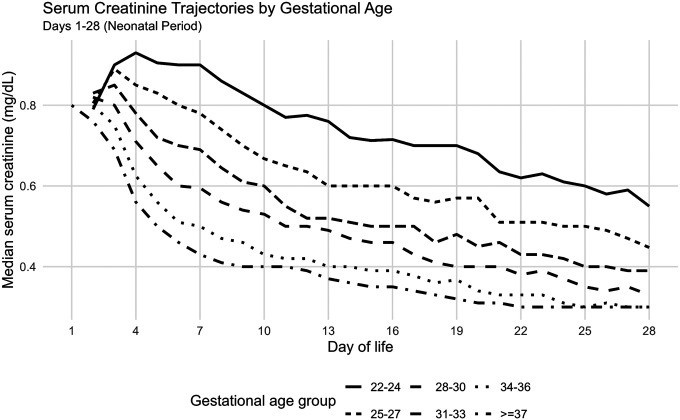
Serum creatinine trajectories by gestational age and day of life (DOL 1–28). Lines represent median serum creatinine values for each gestational age group.

**Figure 2:**
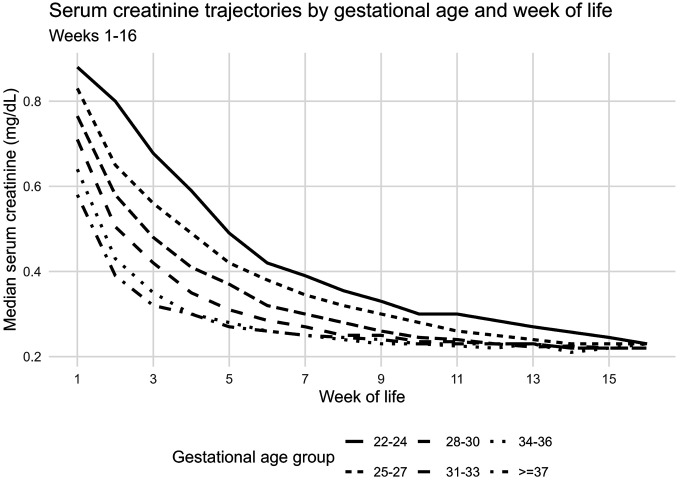
Serum creatinine trajectories by gestational age and week of life (WOL 1–16). Lines represent median serum creatinine values for each gestational age group.

Although our companion interactive tool, NeoKidneyCurve, provides SCr values for each individual GA week, the cohort was stratified into broader GA categories (22–24, 25–27, 28–30, 31–33, 34–36, and ≥ 37 completed weeks) for graphical presentation (Figs. 1 and 2), to allow comparison of SCr trajectories across the spectrum of prematurity without visual overcrowding. Baseline demographic characteristics were summarized at the infant level. Continuous variables, including birth weight and GA at birth, were summarized using medians and interquartile ranges (IQRs), and categorical variables, including sex, maternal Hispanic ethnicity, and maternal race, were summarized as counts and percentages.

Daily trajectory summaries were generated for DOL 1–28, capturing the neonatal period; weekly summaries were generated for WOL 1–16, reflecting the more stable maturational trajectory observed beyond the first month of life. Within each GA group and postnatal time point, the number of contributing infants, median SCr, and IQR were calculated; cells with fewer than 20 contributing infants were suppressed as unreliable. To quantify uncertainty around median SCr estimates, bias-corrected and accelerated (BCa) bootstrapped 95% confidence intervals^20^ were calculated for each median using 1000 replications with the boot^21^ package in R (seed = 42); the percentile bootstrap method was used as a fallback where BCa failed to converge. All analyses were performed using R statistical software version 4.3.0 (R Foundation for Statistical Computing, Vienna, Austria) using the packages dplyr, ggplot2, and gtsummary.^22,23,24^

### Interactive Clinical Tool Development

To facilitate clinical interpretation of SCr values in infants cared for in level IV NICUs, we developed an interactive web-based tool, NeoKidneyCurve, using the Shiny (version 1.7.5) framework in R.^25^ Users enter GA at birth and one or more SCr values with the corresponding DOL at which each was obtained. Values entered beyond DOL 28 are automatically converted to the corresponding WOL for plotting. Based on these inputs, SCr values are displayed against GA-specific median trajectories derived from the cohort, with the estimated percentile of each value relative to infants of the same GA and postnatal time point. Multiple values can be entered simultaneously to visualize longitudinal changes within an individual patient.

When no values have been entered, the landing page displays median SCr trajectories for grouped GA categories by DOL (days 1–28) and WOL (weeks 1–16). After a value is entered, the tool displays the individual GA week’s daily trajectory for values through DOL 28, and the corresponding weekly trajectory extending through the maximum WOL represented in the underlying data (up to WOL 53, reflecting the cohort’s full DOL 1–365 observation window).

## Results

### Study Population

A total of 34,306 infants from ten level IV NICUs met inclusion criteria and were included in the analysis (Table 1). These infants contributed 390,612 SCr measurements obtained between DOL 1 and 365.

Baseline characteristics stratified by GA at birth are shown in Table 1. As expected, median birth weight increased with GA. While Non-Hispanic (NH) White and NH Black infants were consistently the largest groups, the percentage of NH White infants increased with GA, while the percentage of NH Black infants declined.

Aggregation to the infant-day level resulted in 329,233 infant-day observations, with one median SCr value per infant per day. Independent aggregation of the raw SCr data to the infant-week level resulted in 140,780 infant-week observations, with one median value per infant per WOL (Supplemental Figure S1).

### Serum Creatinine Trajectories

SCr trajectories differed substantially by GA at birth across both the neonatal period (DOL 1–28; Fig. 1) and the first 16 weeks of life (Fig. 2). These figures were generated from the same underlying analysis as our companion interactive tool, NeoKidneyCurve, though are presented here as static summary figures rather than direct tool output. Across all GA groups, SCr peaked within the first several days of life then declined, with the timing, peak magnitude, and rate of decline each varying by GA. The most preterm infants (22–24 weeks’ GA) had the highest peak SCr (0.93 mg/dL; DOL 4), followed by those born at 25–27 weeks (0.89 mg/dL; DOL 3) and 28–30 weeks (0.85 mg/dL; DOL 3). Among more mature infants, peak SCr was lower: 0.82 mg/dL at DOL 2 for 31–33 weeks, 0.80 mg/dL at DOL 2 for 34–36 weeks, and 0.76 mg/dL at DOL 2 for ≥ 37 weeks. DOL 1 was excluded from peak identification across all GA groups given very limited sample sizes at this time point (see Limitations).

Following the peak, SCr declined progressively across all GA groups, with the rate of decline most pronounced in the first several weeks of life before gradually plateauing. Observed median SCr values approached a broadly similar nadir of approximately 0.20–0.23 mg/dL across GA groups. The postnatal timing at which this nadir was reached varied across GA but was not consistent across sensitivity analyses excluding a site with limited measurement precision, and did not follow a monotonic pattern across exact GA even in adequately powered strata (Supplemental Table S1); we therefore do not report nadir timing as a precise estimate. The clinical significance of this nadir value, and the mechanisms underlying its timing, warrant further investigation in future studies.

Table 2 summarizes the number of contributing infants, median SCr, and IQR by GA group and DOL (1–28); cells with fewer than 20 contributing infants were suppressed as unreliable rather than reported (see Limitations). Table 3 provides the corresponding summary by GA group and WOL (1–16); all cells in this range were adequately powered (minimum n = 231). Interactive visualization of GA-specific trajectories is available through our companion web-based application, NeoKidneyCurve (https://purl.archive.org/purl/neokidneycurve; Supplemental Figures S2-S4).

## Discussion

### Principal Findings

In this multicenter cohort of infants hospitalized in ten level IV NICUs located in 9 states (AR, DC, NC, OH, WI, TX, IN, GA, MO) from 2010 to 2022, SCr trajectories varied substantially by both GA and postnatal age: SCr rose or remained elevated during the first days of life before declining and eventually stabilizing, with earlier GA associated with higher peak values and a slower, more prolonged decline. These findings highlight the importance of interpreting neonatal SCr in the context of both GA and postnatal age and provide GA-specific longitudinal benchmarks for SCr interpretation in high-acuity NICU populations.

### Physiologic Interpretation of Early Postnatal Creatinine Patterns

The early postnatal patterns observed in this study are consistent with established neonatal kidney physiology. At birth, SCr reflects maternal creatinine levels due to transplacental equilibration, resulting in relatively elevated values during the immediate postnatal period.^8^ As neonatal kidney function matures, glomerular filtration rate increases, leading to a gradual decline in SCr over the first weeks of life.^9^ Preterm infants have reduced nephron number and maturity, lower kidney perfusion, greater tubular permeability leading to backflow of filtered creatinine, and delayed kidney functional development overall.^9,26^ As a result, extremely preterm infants exhibit higher initial SCr values and a more prolonged decline compared with more mature infants. SCr values across all GA groups eventually converged toward a broadly similar nadir (approximately 0.20–0.23 mg/dL); as detailed in the Results, the precise postnatal timing of this convergence varied non-monotonically across GA and was sensitive to site-level measurement precision.

### Comparison with Prior Studies

Prior studies describing postnatal SCr patterns, particularly among preterm infants within restricted GA ranges, have more commonly been single-center,^12–14^ and many have relied on cross-sectional measurements rather than longitudinal trajectories. Differences in laboratory measurement methods — including the use of modified Jaffe versus enzymatic assays — further complicate direct comparisons across studies, as SCr values are expected to vary by as much as 30% around the mean based on both patient and assay factors.^27^ A recent study using advanced modeling techniques characterized SCr and creatinine clearance dynamics in 148 extremely low birth weight infants, providing valuable model-based insight into this population;^28^ our study extends this work using an empirical, percentile-based approach across a substantially larger cohort (34,306 infants) spanning the full range of GA from 22 to 42 weeks, rather than a single narrow GA stratum. This approach provides a more comprehensive description of SCr trajectories across the neonatal period for infants admitted to level IV NICUs and additional context for interpreting SCr values in high-acuity populations.

### Clinical Implications and Role of the Interactive Tool

Interpretation of SCr in infants is complex, as values vary substantially by both GA and postnatal age. A single SCr threshold may therefore be misleading; values that are expected for an extremely preterm infant in early postnatal life may be atypical in a more mature infant.^9,27^ The GA-specific longitudinal trajectories described in this study provide a framework for interpreting SCr values and may assist clinicians in distinguishing expected physiologic patterns from values that fall outside this expected range.

Trajectory-based interpretation may support earlier recognition of impaired kidney function, which is critical given that neonatal AKI is common in the NICU and associated with increased morbidity and mortality.^30^ Infants with SCr values persistently exceeding higher percentile thresholds (e.g., > 75th percentile) may warrant closer clinical evaluation. Chen et al. demonstrated that infants with SCr above the 95th percentile for postnatal age had mortality rates comparable to those meeting neonatal KDIGO AKI criteria, underscoring the clinical relevance of percentile-based interpretation.^31^ Such context may be particularly relevant during high-risk clinical states — including sepsis, patent ductus arteriosus treatment, necrotizing enterocolitis, extracorporeal membrane oxygenation support, nephrotoxic medication exposure, and postoperative care — during which kidney function often changes rapidly.

Previous studies have demonstrated that postnatal creatinine trajectories may provide clinically relevant information beyond isolated creatinine values.^32–36^ In preterm neonates specifically, a delayed rate of SCr decline has been associated with longer length of stay, prolonged ventilation, and greater need for vasoactive medications and diuretics.^36^ Our findings extend this concept by providing GA-specific longitudinal trajectories across a large multicenter cohort of high-acuity NICU infants.

Recognition of atypical SCr trajectories may help alert clinicians to potential kidney injury or impaired function, potentially prompting renoprotective strategies such as optimization of kidney perfusion through maintenance of adequate blood pressure, careful fluid management, avoidance of or reductions in nephrotoxic medications, and closer monitoring of kidney function.^37,38,39^ In this context, trajectory-based interpretation may prompt consideration of clinically actionable steps, including more frequent SCr monitoring, tighter fluid balance, medication review, and early nephrology consultation.

Rather than functioning solely as a static laboratory measurement, neonatal SCr may serve as a dynamic developmental biomarker reflecting the interaction between renal maturation, nephron developmental stage, systemic illness severity, and evolving hemodynamic adaptation. To facilitate clinical application of these findings, we developed an interactive web-based tool, NeoKidneyCurve, that allows users to visualize individual SCr values relative to GA-specific trajectories and view the corresponding percentile for an infant’s GA and postnatal age. The trajectories presented in this study provide GA- and postnatal age-specific context for interpreting SCr values in infants admitted to level IV NICUs. Future studies are needed to determine whether incorporation of these trajectories into clinical workflows improves recognition of kidney dysfunction, clinical decision-making, or patient outcomes. Separately, structured nephrotoxin-monitoring and fluid-stewardship bundles have demonstrated improvements in kidney-related outcomes in NICU populations;^40^ whether trajectory-based context of the kind presented here can support similar structured approaches is an important question for future work.

To our knowledge, NeoKidneyCurve represents one of the largest publicly available, multicenter resources for GA-specific SCr interpretation in critically ill neonates, built from 34,306 infants across ten level IV NICUs. The tool is freely accessible online, requires no software installation, and allows clinicians to enter a patient’s GA and one or more SCr values to instantly visualize that patient’s trajectory against the full cohort, at either daily or weekly resolution depending on postnatal age. For each value entered, the tool reports the corresponding percentile for that infant’s exact GA and postnatal day or week, allowing clinicians to directly contextualize an individual SCr value relative to a large cohort of other critically ill NICU infants of the same maturity and postnatal age, rather than relying on a single fixed threshold. By translating a large, complex multicenter dataset into an interactive, bedside-accessible format, this work aims to make GA- and postnatal age-specific context readily available at the point of care, rather than requiring clinicians to consult static tables or published literature.

## Limitations

Several limitations should be considered. This study was conducted in level IV NICUs among infants with complex medical conditions, including congenital anomalies, surgical conditions, and critical illness. AKI and critical illness were intentionally not exclusionary, as our objective was to characterize a real-world level IV NICU population rather than to construct a healthy-infant reference standard. Accordingly, these trajectories should be interpreted as patterns observed in a high-acuity NICU population rather than as normative values for healthy infants, and may not generalize to lower-acuity settings. Because SCr measurements were obtained through routine clinical care rather than a standardized protocol, infants with greater illness severity likely contributed disproportionately more observations, introducing potential surveillance bias, and measurement frequency and timing varied across patients and centers.

DOL 1 was excluded from peak SCr identification across all GA groups due to very limited sample sizes at this time point; similarly, DOL 1 was the only time point suppressed in Table 2 due to fewer than 20 contributing infants, with all other cells across days 2 through 28 adequately powered. SCr assay methodology was not captured uniformly across ADVANCE sites and may have varied within sites over the study period, introducing inter- and intra-center variability in absolute SCr values. Consistent with this, one site reported SCr values exclusively to one decimal place, and another site’s data contained no values below 0.2 mg/dL, suggesting that the observed convergence in SCr values across GA groups may partly reflect measurement or reporting resolution rather than a purely physiologic steady state. Finally, SCr is an imperfect biomarker of kidney function, influenced by factors including muscle mass, fluid status, and maternal creatinine levels.^9^ The trajectories and interactive tool were developed using a single multicenter database and have not yet undergone external validation in independent neonatal populations.

## Conclusions

In this large multicenter cohort of infants hospitalized in level IV NICUs, SCr trajectories varied substantially by GA and postnatal age. GA-specific longitudinal trajectories provide clinically useful context for interpreting SCr values in high-acuity neonatal populations. The accompanying interactive tool offers a practical approach for applying these data at the bedside. Future studies are needed to evaluate the applicability of these trajectories in broader neonatal populations and to assess their role in improving clinical decision-making and outcomes. Future work should focus on integration into electronic health record–based clinical decision support systems and incorporation of complementary kidney metrics, including urine output, fluid balance, and nephrotoxic medication exposure burden.

## Supplementary Material

Tables 2 and 3

Tables 2 and 3 are available in the Supplementary Files section.

Supplementary Files

This is a list of supplementary files associated with this preprint. Click to download.

- supplementaboutapp.png
- supplementlanding.png
- Table2DOLSCr.rtf
- Table3WOLSCr.rtf
- SupplementalTableS1Nadir.rtf
- Table1TrajectoriesDemographics.docx
- trajectoriescohortflowcolor.png
- supplementcalculator.png

## Figures and Tables

**Table 1:** Characteristics of the Study Population by Gestational Age Group

Characteristic	Overall, N = 34,306^1^	22–24, N = 1,859^1^	25–27, N = 2,960^1^	28–30, N = 2,498^1^	31–33, N = 3,073^1^	34–36, N = 6,346^1^	>=37, N = 17,570^1^
Birth weight, g	2,661 (1,540–3,290)	615 (543–697)	840 (705–970)	1,220 (1,005–1,430)	1,770 (1,488–2,070)	2,490 (2,125–2,875)	3,220 (2,845–3,600)
Gestational age at birth, weeks	37.0 (32.1–39.0)	24.0 (23.4–24.4)	26.3 (25.6–27.0)	29.3 (28.6–30.1)	32.4 (31.9–33.3)	35.7 (34.9–36.3)	39.0 (38.0–39.7)
Sex							
Female	14,704 (42.9%)	885 (47.6%)	1,306 (44.1%)	1,070 (42.8%)	1,356 (44.1%)	2,688 (42.4%)	7,399 (42.1%)
Male	19,572 (57.1%)	972 (52.3%)	1,653 (55.8%)	1,426 (57.1%)	1,712 (55.7%)	3,656 (57.6%)	10,153 (57.8%)
Other/Indeterminate	28 (0.1%)	2 (0.1%)	0 (0.0%)	1 (0.0%)	5 (0.2%)	2 (0.0%)	18 (0.1%)
Unknown/Missing	2 (0.0%)	0 (0.0%)	1 (0.0%)	1 (0.0%)	0 (0.0%)	0 (0.0%)	0 (0.0%)
Hispanic ethnicity							
No	28,817 (84.0%)	1,611 (86.7%)	2,528 (85.4%)	2,153 (86.2%)	2,658 (86.5%)	5,333 (84.0%)	14,534 (82.7%)
Unknown/Missing	1,903 (5.5%)	80 (4.3%)	130 (4.4%)	109 (4.4%)	129 (4.2%)	356 (5.6%)	1,099 (6.3%)
Yes	3,586 (10.5%)	168 (9.0%)	302 (10.2%)	236 (9.4%)	286 (9.3%)	657 (10.4%)	1,937 (11.0%)
Maternal race							
American Indian or Alaska Native	105 (0.3%)	1 (0.1%)	6 (0.2%)	14 (0.6%)	17 (0.6%)	30 (0.5%)	37 (0.2%)
Asian	1,112 (3.2%)	61 (3.3%)	76 (2.6%)	55 (2.2%)	81 (2.6%)	175 (2.8%)	664 (3.8%)
Black	8,917 (26.0%)	857 (46.1%)	1,157 (39.1%)	819 (32.8%)	885 (28.8%)	1,345 (21.2%)	3,854 (21.9%)
Native Hawaiian or Pacific Islander	50 (0.1%)	5 (0.3%)	3 (0.1%)	0 (0.0%)	7 (0.2%)	10 (0.2%)	25 (0.1%)
Other	2,762 (8.1%)	120 (6.5%)	213 (7.2%)	177 (7.1%)	215 (7.0%)	493 (7.8%)	1,544 (8.8%)
Unknown/Missing	1,902 (5.5%)	84 (4.5%)	123 (4.2%)	121 (4.8%)	123 (4.0%)	337 (5.3%)	1,114 (6.3%)
White	19,458 (56.7%)	731 (39.3%)	1,382 (46.7%)	1,312 (52.5%)	1,745 (56.8%)	3,956 (62.3%)	10,332 (58.8%)

1 Median (25%−75%); n (%)

Values are median (interquartile range) or n (%).

## Data Availability

Data may not be shared due to institutional and data use agreements.

## Abbreviations

AKI: Acute Kidney Injury
DOL: Day of Life
WOL: Week of Life
NICU: Neonatal Intensive Care Unit

## References

[1] Go H, Momoi N, Kashiwabara N, Haneda K, Chishiki M, Imamura T Neonatal and maternal serum creatinine levels during the early postnatal period in preterm and term infants. PLoS One 2018; 13: e0196721. 29795567 10.1371/journal.pone.0196721 PMC5967735

[2] Gubhaju L, Sutherland MR, Horne RSC, Medhurst A, Kent AL, Ramsden A Assessment of renal functional maturation and injury in preterm neonates during the first month of life. Am J Physiol Renal Physiol 2014; 307: F149–F158. 24899060 10.1152/ajprenal.00439.2013

[3] Smeets NJL, IntHout J, van der Burgh MJP, Schwartz GJ, Schreuder MF, de Wildt SN. Maturation of GFR in term-born neonates: an individual participant data meta-analysis. J Am Soc Nephrol 2022; 33: 1277–1292. 35474022 10.1681/ASN.2021101326 PMC9257816

[4] Gallini F, Maggio L, Romagnoli C, Marrocco G, Tortorolo G. Progression of renal function in preterm neonates with gestational age ≤ 32 weeks. Pediatr Nephrol 2000; 15: 119–124. 11095027 10.1007/s004670000356

[5] Kastl JT. Renal function in the fetus and neonate – the creatinine enigma. Semin Fetal Neonatal Med 2017; 22: 83–89. 28109705 10.1016/j.siny.2016.12.002

[6] Hinchliffe SA, Sargent PH, Howard CV, Chan YF, van Velzen D. Human intrauterine renal growth expressed in absolute number of glomeruli assessed by the disector method and Cavalieri principle. Lab Invest 1991; 64: 777–784. 2046329

[7] Sutherland MR, Gubhaju L, Moore L, Kent AL, Dahlstrom JE, Horne RSC Accelerated maturation and abnormal morphology in the preterm neonatal kidney. J Am Soc Nephrol 2011; 22: 1365–1374. 21636639 10.1681/ASN.2010121266 PMC3137584

[8] Guignard JP, Drukker A. Why do newborn infants have a high plasma creatinine? Pediatrics 1999; 103: e49. 10103341 10.1542/peds.103.4.e49

[9] Jetton JG, Askenazi DJ. Acute kidney injury in the neonate. Clin Perinatol 2014; 41: 487–502. 25155722 10.1016/j.clp.2014.05.001

[10] Mohr Lytsen R, Taageby Nielsen S, Kongsgaard Hansen M, Strandkjær N, Juul Rasmussen I, Axelsson Raja A Markers of kidney function in early childhood and association with maternal comorbidity. JAMA Netw Open 2022; 5: e2243146. 36409493 10.1001/jamanetworkopen.2022.43146 PMC9679880

[11] Bueva A, Guignard JP. Renal function in preterm neonates. Pediatr Res 1994; 36: 572–577. 7877873 10.1203/00006450-199411000-00005

[12] Askenazi DJ, Ambalavanan N, Hamilton K, Cutter G, Laney D, Kaslow R Acute kidney injury and renal replacement therapy independently predict mortality in neonatal and pediatric noncardiac patients on extracorporeal membrane oxygenation. Pediatr Crit Care Med 2011; 12: e1–e6. 20351617 10.1097/PCC.0b013e3181d8e348

[13] Bateman DA, Thomas W, Parravicini E, Polesana E, Locatelli C, Lorenz JM. Serum creatinine concentration in very-low-birth-weight infants from birth to 34–36 weeks postmenstrual age. Pediatr Res 2015; 77: 696–702. 25675426 10.1038/pr.2015.25 PMC4407015

[14] Jetton JG, Boohaker LJ, Sethi SK, Bhatt GC, Steflik HJ, Bhatt P Incidence and outcomes of neonatal acute kidney injury (AWAKEN): a multicenter, multinational, observational cohort study. Lancet Child Adolesc Health 2017; 1: 184–194. 29732396 10.1016/S2352-4642(17)30069-X PMC5933049

[15] Boer DP, de Rijke YB, Hop WC, Cransberg K, Dorresteijn EM, van Wijk JA. Reference values for serum creatinine in children younger than 1 year of age. Pediatr Nephrol 2010; 25: 2107–2113. 20505955 10.1007/s00467-010-1533-y PMC2923720

[16] Chuang GT, Tsai IJ, Tsau YK. Establishing a reference range for serum creatinine in the neonatal population and re-defining neonatal acute kidney injury. Pediatr Nephrol 2026; 41: 1525–1534. 41504897 10.1007/s00467-025-07141-1

[17] Committee on Fetus and Newborn; Barfield WD, Papile L-A, Baley JE, Benitz W, Cummings J Levels of neonatal care. Pediatrics 2012; 130: 587–597. 22926177 10.1542/peds.2012-1999

[18] Rumpel JA, Perazzo S, Bona J, South AM, Harer MW, Liu D ADVANCE: a biomedical informatics approach to investigate acute kidney injury in infants. Pediatr Res 2025; 97: 608–613. 39122822 10.1038/s41390-024-03436-5 PMC12024515

[19] Murthy K, Dykes FD, Padula MA, Pallotto EK, Reber KM, Durand DJ The Children’s Hospitals Neonatal Database: an overview of patient complexity, outcomes and variation in care. J Perinatol 2014; 34: 582–586. 24603454 10.1038/jp.2014.26

[20] Efron B. Better bootstrap confidence intervals. J Am Stat Assoc 1987; 82: 171–185.

[21] Canty A, Ripley BD. boot: Bootstrap R (S-Plus) Functions. R package version 1.3–28.1; 2022. Available at: https://CRAN.R-project.org/package=boot

[22] R Core Team. R: A language and environment for statistical computing. R Foundation for Statistical Computing: Vienna, Austria, 2023.

[23] Wickham H. ggplot2: Elegant Graphics for Data Analysis. Springer: New York, NY, 2016.

[24] Sjoberg DD, Whiting K, Curry M, Lavery JA, Larmarange J. Reproducible summary tables with the gtsummary package. R J 2021; 13: 570–580.

[25] Chang W, Cheng J, Allaire JJ, Sievert C, Schloerke B, Xie Y shiny: Web Application Framework for R. R package; 2023. Available at: https://CRAN.R-project.org/package=shiny

[26] Fawer CL, Torrado A, Guignard JP. Maturation of renal function in full-term and premature neonates. Helv Paediatr Acta 1979; 34: 11–21. 429191

[27] Allegaert K, Kuppens M, Mekahli D, Levtchenko E, Vanstapel F, Vanhole C Creatinine reference values in ELBW infants: impact of quantification by Jaffe or enzymatic method. J Matern Fetal Neonatal Med 2012; 25: 1678–1681. 22273037 10.3109/14767058.2012.657277

[28] van Donge T, Allegaert K, Gotta V, Smits A, Levtchenko E, Mekahli D Characterizing dynamics of serum creatinine and creatinine clearance in extremely low birth weight neonates during the first 6 weeks of life. Pediatr Nephrol 2021; 36: 649–659. 32944826 10.1007/s00467-020-04749-3 PMC7851041

[29] Askenazi DJ, Griffin R, McGwin G, Carlo W, Ambalavanan N. Acute kidney injury is independently associated with mortality in very low birthweight infants: a matched case-control analysis. Pediatr Nephrol 2009; 24: 991–997. 19238451 10.1007/s00467-009-1133-x

[30] Selewski DT, Charlton JR, Jetton JG, Guillet R, Mhanna MJ, Askenazi DJ Neonatal acute kidney injury. Pediatrics 2015; 136: e463–e473. 26169430 10.1542/peds.2014-3819

[31] Chen C-C, Lin Y-C, Huang C-C. The overlooked subgroup: preterm neonates with elevated serum creatinine outside neonatal AKI criteria. Pediatr Res 2025. 10.1038/s41390-025-04365-7 40962862

[32] Gupta C, Massaro AN, Ray P. A new approach to define acute kidney injury in term newborns with hypoxic ischemic encephalopathy. Pediatr Nephrol 2016; 31: 1167–1178. 26857710 10.1007/s00467-016-3317-5 PMC4882244

[33] Ahn HC, Frymoyer A, Boothroyd DB, Bonifacio S, Sutherland SM, Chock VY. Acute kidney injury in neonates with hypoxic ischemic encephalopathy based on serum creatinine decline compared to KDIGO criteria. Pediatr Nephrol 2024; 39: 2789–2796. 38326648 10.1007/s00467-024-06287-8

[34] Rumpel JA, Spray BJ, Frymoyer A, Rogers S, Cho SH, Ranabothu S Renal oximetry for early acute kidney injury detection in neonates with hypoxic ischemic encephalopathy receiving therapeutic hypothermia. Pediatr Nephrol 2023; 38: 2839–2849. 36786860 10.1007/s00467-023-05892-3

[35] Chock VY, Frymoyer A, Yeh CG, Van Meurs KP. Renal saturation and acute kidney injury in neonates with hypoxic ischemic encephalopathy undergoing therapeutic hypothermia. J Pediatr 2018; 200: 232–239. 29866591 10.1016/j.jpeds.2018.04.076

[36] Perazzo S, Revenis M, Massaro A, Short BL, Ray PE. A new approach to recognize neonatal impaired kidney function. Kidney Int Rep 2020; 5: 2301–2312. 33305124 10.1016/j.ekir.2020.09.043 PMC7710891

[37] Pandey V, Kumar D, Vijayaraghavan P, Chaturvedi T, Raina R. Non-dialytic management of acute kidney injury in newborns. J Renal Inj Prev 2016; 6: 1–11. 28487864 10.15171/jrip.2017.01 PMC5414511

[38] Hanna MH, Askenazi DJ, Selewski DT. Drug-induced acute kidney injury in neonates. Curr Opin Pediatr 2016; 28: 180–187. 26735892 10.1097/MOP.0000000000000311 PMC4824298

[39] Branagan A, Costigan CS, Stack M, Slagle C, Molloy EJ. Management of acute kidney injury in extremely low birth weight infants. Front Pediatr 2022; 10: 867715. 35433560 10.3389/fped.2022.867715 PMC9005741

[40] Askenazi DJ, Gordon L, Griffin R, Halloran B, Ambalavanan N, Garg PM Reducing NICU ventilator days by preventing fluid overload with the CAN-U-P-LOTS standardized bundle. Pediatr Res 2026; 99: 889–897. 40646283 10.1038/s41390-025-04078-x PMC13021496

